# Comparison of decayed, missing, filled teeth index between thalassemia major patients and control group in Iran: a systematic review and meta-analysis

**DOI:** 10.1038/s41405-020-00051-4

**Published:** 2020-11-17

**Authors:** Mahmood Moosazadeh, Nadia Elyassi Gorji, Pegah Nasiri, Ali Malekzadeh Shafaroudi

**Affiliations:** 1 Gastrointestinal Cancer Research Center, Non-communicable Diseases Institute, Mazandaran University of Medical Sciences, Sari, Iran; 2 Health Science Research Center, Addiction Institute, Mazandaran University of Medical Sciences, Sari, Iran; 3 Student Research Committee, Faculty of Dentistry, Mazandaran University of Medical Sciences, Sari, Iran

**Keywords:** Dental public health, Dental treatments

## Abstract

**Introduction:**

Scientific evidence of the association between Decayed, Missing, and Filled Teeth (DMFT) and thalassemia are conflicting and difficult to establish conclusions. Therefore, this study aimed to determine the relationship between dental caries (using the DMFT index) and thalassemia major using meta-analysis.

**Methods:**

Databases were searched using such keywords as “Thalassemia,” “Caries,” “decay,” “DMFT,” “Iran,” and OR operators, AND, and NOT. After the elimination of duplicate documentation, the articles which met the inclusion criteria were selected. Quality assessment was performed based on the Newcastle-Ottawa Quality Checklist. Thereafter, the standardized mean difference of the DMFT index was estimated.

**Results:**

In eight studies, the mean DMFT was compared between patients with thalassemia major and the control group. In six studies, the mean of this index was higher in patients with thalassemia major than in the control group and in all six of the studies, the differences were statistically significant. The mean standardized difference of DMFT, D, M, and F were reported as 1.36 (0.41, 2.30), 2.63 (0.42, 4.84), 1.65 (−0.14, 3.45), and 0.02 (−1.67, 1.72), respectively.

**Conclusion:**

The results of this meta-analysis indicated that DMFT index was more inappropriate in patients with thalassemia, as compared to the control group which represents the higher incidence of dental caries among patients with thalassemia compared to the control group.

## Introduction

Major thalassemia is an autosomal recessive blood disease and a type of congenital hemolytic anemia whose genetic defects can be in the form of point mutations or deletion or a combination of globin genes.^1^ Thalassemia is classified into two types: alpha-thalassemia and beta-thalassemia. Alpha-thalassemia is a hemoglobin genetic abnormality that is caused by reduced production of the alpha-globin chains.^2^ The alpha chain is present in both adult and fetal hemoglobin, which deficiency makes the synthesis of both types of hemoglobin difficult.^3^ Beta-thalassemia is the most common form of thalassemia in which the production of the beta chain is reduced.^4^ The highest prevalence of the disease has been seen in Mediterranean countries, parts of the north and west of Africa, the Middle East region, and the Far East countries, all of which are located on the Thalassemia belt.^5^

Iran is one of the thalassemia generate countries in the world, according to WHO, about 4% of Iranians carry the thalassemia gene and more than 18,000 cases of thalassemia are scattered all across the country.^6^ Patients with thalassemia have no specific clinical symptoms at birth but gradually develop symptoms from the age of 3 to 6 months and as the gamma chain of hemoglobin is transmitted to beta, the patient will develop anemia; therefore, require daily blood transfusions to survive.^7,8^ Despite improving oxygen transfer to tissues, suppression of ineffective hematopoiesis, and prolonging the patient lifespan repeated blood transfusions is followed by common complications such as endocrine defects, risk of viral infections (such as hepatitis B, hepatitis C, HIV, etc.), and accumulation of iron in the body.^9^ Nowadays, a major shift has occurred in the treatment of these patients, with therapeutic advances, especially after the initiation of iron-chelating treatment such as desferal or deferoxamine injection. The use of these agents has increased the life expectancy of patients.^10^

One of the subcategories of health-related quality of life is oral health-related quality of life;^11^ therefore, oral diseases can affect the quality of life, and oral health and general health are not separated from each other.^12^ Oral and maxillofacial problems such as dental caries and gingival diseases are one of the major concerns of patients with thalassemia. According to a 2013 study, the prevalence of dental caries and periodontal diseases in patients with thalassemia was higher than in the control group.^13^ The teeth of people with thalassemia undergo morphological changes, most notably a reduction of teeth size including a reduction in mesio-distal width of teeth, and an increase in the number of pits and fissures that, together with endocrine dysfunction and poor oral health, increase the risk of tooth decay.^14,15^ Some studies believe endocrine problems, malocclusion and poor motivation of patients are responsible for the increase of tooth decay in patients with thalassemia.^16,17^

A relatively significant number of preliminary studies have examined the link between thalassemia and the DMFT index, which also suggests inconsistencies. One of the solutions to combine the results of preliminary studies and resolve contradictions and provide an overall estimate with larger sample size and higher test power is to use meta-analysis. As far as the authors are aware, there is no evidence of meta-analysis conducted on this subject. Accordingly, this study aimed to compare the DMFT index of patients with thalassemia major with the control group using meta-analysis.

## Material and methods

### Search strategy

Inclusion and exclusion criteria were set according to the PICO approach. In this study, P included patients with thalassemia major, I was not associated with the type of study since the study did not involve any intervention, C included people who did not have thalassemia major and were considered as the control group, O included DMFT, D, F and M indicators in the group of patients with thalassemia major and the control group. The type of study included case-control and cross-sectional studies. This study aimed to compare the DMFT of patients with thalassemia major with the control group.

### Data sources

The databases PubMed, Scopus, Science direct, web of science and Magiran and SID Iranian databases were searched between 1 December 2019 and 29 April 2020 using Mesh and non-mesh keywords such as “thalassemia,” “DMFT,” “meta-analysis,” “decay,” “caries”, and “Iran” and OR, AND and NOT operators were placed.

All of the initial searched articles were imported to the Endnote software where the duplicate titles searched from all of the databases and search engines by each of the two authors were excluded. All articles in English and Persian were carefully reviewed and articles in other languages were also excluded from this meta-analysis.

The list of references for all searched articles was also reviewed to increase the sensitivity of the search and to select more studies.

### Eligibility criteria and article selection

The screening process of the articles was done by reviewing the title and abstract of the initial studies. Thus, unrelated articles were removed by reviewing the title and abstract. The full text of the articles was received to comply with the entry and exit criteria. This process was performed by two people. Disputes between the two were discussed. Published studies from any time until the end of January 2020 have been among the eligibility criteria. Articles without full text and efficient data, data listed in invalid studies such as thesis which were not published until the date of search of this study were excluded.

### Study quality assessment

To assess the quality of the qualified articles, The Newcastle-Ottawa Scale checklist was used. This checklist includes three sections: Selection, Comparability, and Exposure. The checklist score is between 9-0. The Selection criterion has a maximum of four scores, the Comparability criterion has a maximum of two scores, and the Exposure criterion has a maximum of 3 scores.^18^ Evaluations were provided independently by two people. Articles with scores below 5 were excluded from the study.

### Data extraction

Data were extracted independently by two authors. Three groups of variables were extracted from the initial studies. The first group of identification variables included the first author’s last name, article title, journal name, year of publication, place of study, and language of publication. The second group of variables was related to sample size in the group of patients with thalassemia major, the sample size in the control group, the mean and standard deviation of DMFT, D, F, and M parameters. The third group included the variables needed to evaluate the Risk of Bias and focused on methodology, including how to select cases and controls, method of matching the case and control groups based on age and sex, and evaluation of DMFT, D, F, and M in both groups.

### Statistical analysis methods

The Stata software ver. 11 was used to analyze the data. The heterogeneity index for individual studies was determined using the Cochrane test (Q) and *I*^2^ tests. The random-effect model was used to estimate the standardized difference between the mean parameters of DMFT, D, F, and M in the group of patients with thalassemia major compared to the control group. The method used for estimation was the reverse variance method and Cohen’s statistic.

The standardized point mean difference estimate of the average performance score of DMFT, D, F, and M indicators were presented with a 95% confidence interval as forest plots, in which the square size represents the weight of each study and the lines on either side of it show a 95% confidence interval. In cases where the confidence interval did not include zero, the observed difference was considered statistically significant. The effect of each initial study on the overall estimate was analyzed by sensitivity analysis.

## Results

**Fig. 1:**
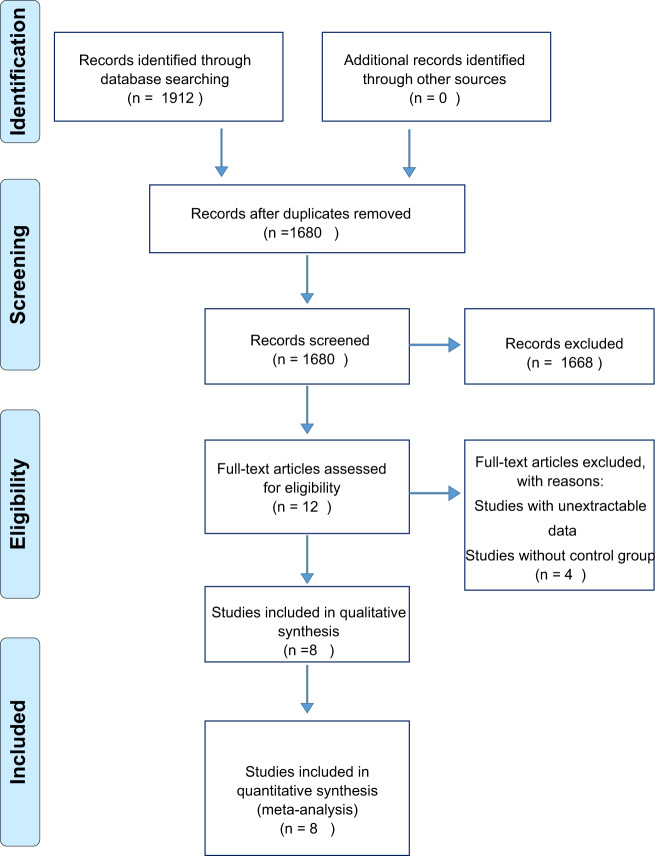
Flow diagram of the systematic review design process.

The search strategy included the assessment of article titles, abstracts, and keywords. The initial search in seven databases based on the proper keywords resulted in 1912 articles. After the primary screening and removing duplicate documents that overlapped in the databases, 1680 articles were selected. Next, studies without full text were excluded and after screening based on title and abstract, 1641 unrelated articles were identified and excluded because ether their data was not extractable or because they lacked a control group. Finally, the quality assessment of 39 articles was performed, and 8 articles were included in the meta-analysis (Fig. 1).

**Fig. 2:**
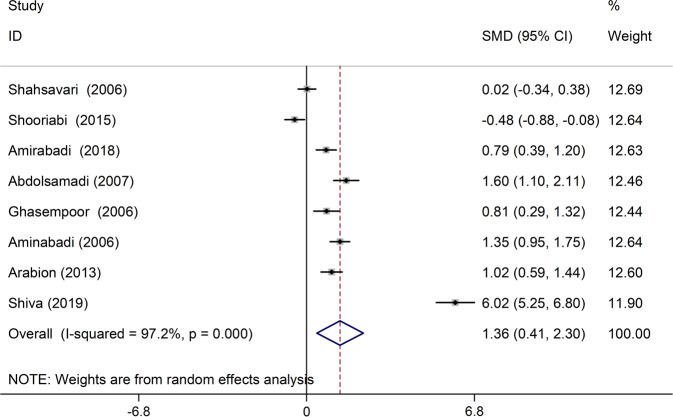
The standardized mean difference of forest plots of the DMFT index in each primary study and the overall estimate.

**Fig. 3:**
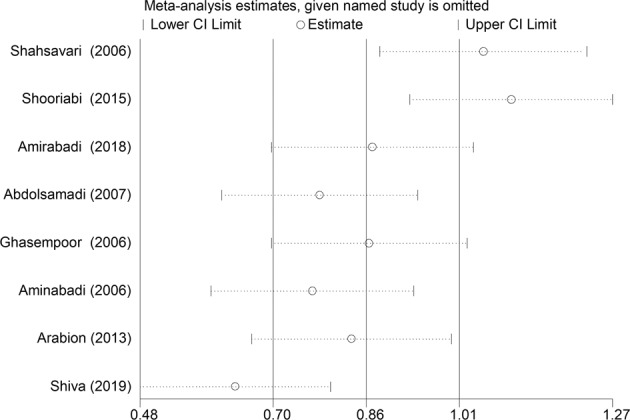
Sensitivity analysis results to evaluate the impact of each study on standardized mean difference estimate.

In eight studies, the mean DMFT was compared between patients with thalassemia major and the control group (Table 1). In six studies, the mean of this index in patients with thalassemia major was higher than the control group, and in all six of these studies, the observed differences were statistically significant^19–24^ Also, in one study, the mean DMFT in the group of patients with thalassemia major was significantly lower than in the control group.^25^ The results of heterozygous indicators showed that the discrepancy between the initial studies was significant (I-squared:97.2%, *Q* = 250.97, *P* < 0.001). Combining the results of preliminary studies based on the random effect model, it was shown that the average standardized difference (CI 95%: 0.41, 2.30) is 1.36 (Fig. 2). Also, based on the results of sensitivity analysis, the effect of each of the initial studies on the overall estimate was not significant (Fig. 3).

**Table 1:** Characteristics of primary studies included in the meta-analysis.

First author, publication year	Area study	Type study	Case (Thalassemia)-DMFT			Control-DMFT			The score of quality assessment
			Sample size	Mean	SD	Sample size	Mean	SD	
Shahsavari, 2006^39^	gilan	cross sectional	60	7.33	3.80	60	7.26	3.73	6
Shooriabi, 2015^25^	ahvaz	cross sectional	50	4.94	1.5	50	5.8	2.04	8
Amirabadi, 2018^19^	zahedan	cross sectional	50	5.360	2.577	50	3.360	2.456	7
Abdolsamadi, 2007^20^	hamedan	historical cohort	28	7.35	3.05	60	3.38	2.16	6
Ghasempoor, 2006^21^	babol	case control	31	5.26	4.16	31	2.65	1.91	6
amin abadi, 2006^22^	tabriz	analytic descriptive	60	6.5	3.24	60	2.8	2.13	6
Arabion, 2013^23^	Shiraz	case-control	47	6.45	1.63	48	4.86	1.5	7
Shiva, 2019^24^	Sari	Cross-sectional	75	4.67	0.234	69	3.30	0.22	6

**Fig. 4:**
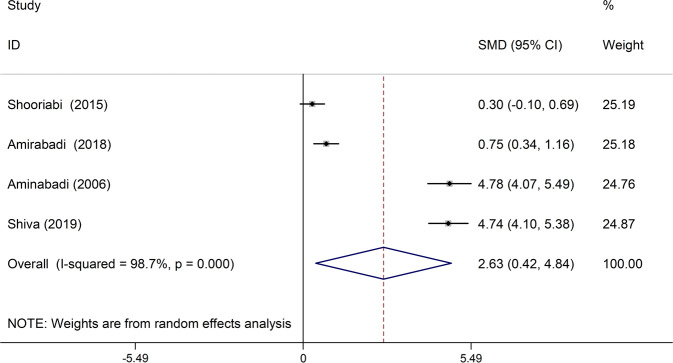
The standardized mean difference of forest plots of D index in each primary study and the overall estimate.

In four studies, the mean D index was compared between the case group and the control group. In all four studies, the mean of this index in the case group was higher than the control group,^19,22,24,25^ which was statistically significant in three studies.^19,22,24^ There was also a significant discrepancy between the studies (I-squared: 98.71%, *Q*: 228.80, *P* < 0.001). Combining the results of these four studies, the average standardized difference (CI 95%: 0.42, 4.84) was estimated to be 2.63 (Fig. 4).

**Fig. 5:**
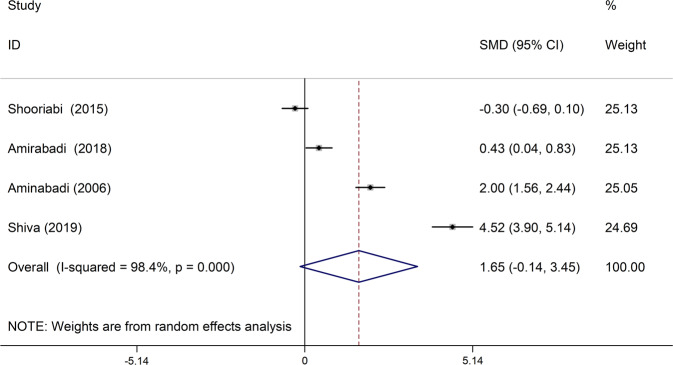
The standardized mean difference of forest plots of M index in each primary study and the overall estimate.

In four studies, the mean of the M index was compared between the case group and the control group. In three of these studies, the mean of this index in the case group was higher than the control group, which was statistically significant in all three studies.^19,22,24^ There was also a significant discrepancy between the studies (I-squared: 98.4%, *Q*: 192.43, *P* < 0.001). Combining the results of these four studies, the average standardized difference (CI 95%: −0.14, 3.45) was estimated to be 1.65 (Fig. 5).

**Fig. 6:**
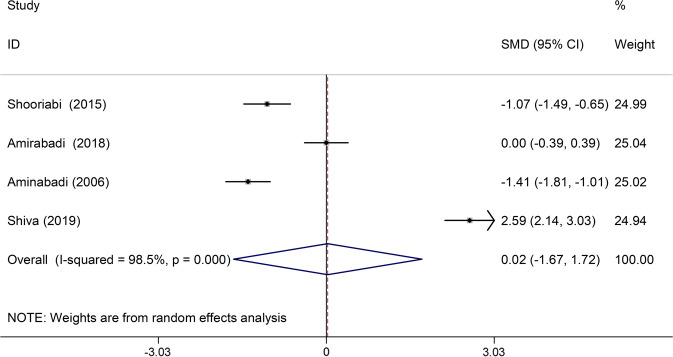
The standardized mean difference of forest plots of F index in each primary study and the overall estimate.

In four studies, the mean of the F index was compared between the case group and the control group. In two studies, the mean of this index in the case group was less than the control group, which in both studies was statistically significant.^24,25^ There was also a significant discrepancy between the studies (I-squared: 98.5%, *Q*: 202.44, *P* < 0.001). Combining the results of these four studies, the average standardized difference (CI 95%: −1.67, 1.72) was estimated to be 0.02 (Fig. 6).

## Discussion

In the present study, the results of preliminary studies were combined to investigate the relationship between DMFT index and thalassemia major using meta-analysis. The study found that the mean standardized DMFT difference in the thalassemia major group was significantly 1.36 units higher than the control group. Also, the D and M indexes in the thalassemia major group were 2.63 and 1.65 units higher than the control group, respectively. However, this difference was not statistically significant. It should be noted that the F index in the thalassemia major group was 0.02 units lower than the control group, which was not statistically significant.

**Fig. 7:**
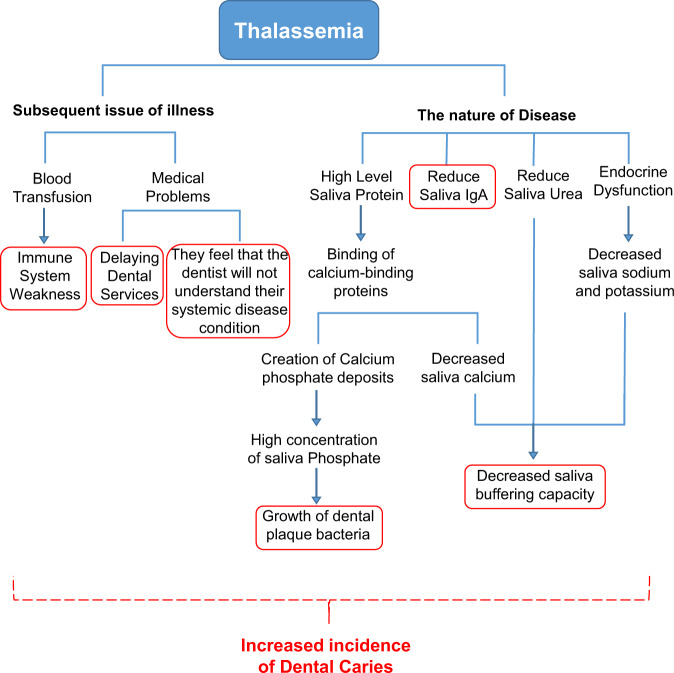
Effects of thalassemia on dental caries.

Al-Wahdani stated that there was no clear link between thalassemia major and periodontal disease, but that the prevalence of caries increased significantly in these patients.^26^ Siamopoulou also found that the reduction of IgA in the saliva and increase of proliferation of oral bacteria was effective in increasing the DMFT of these patients.^27^ Buczkowska and Lopez, meanwhile, claimed that low calcium concentrations in the saliva were effective in increasing the rate of tooth decay. As the high protein content of saliva results in the binding of calcium-binding proteins and the formation of calcium phosphate deposits adjacent to the teeth.^28,29^ Also, Pandey,^30^ Gandhy,^31^ and Bardow^32^ considered high concentrations of saliva phosphate to be effective in causing dental caries. Luglie also showed that reducing salivary urea in patients with thalassemia was effective in increasing the prevalence of caries.^33^ Kalman et al. also claimed that the levels of sodium, potassium, and calcium in saliva decreased significantly in people with thalassemia major compared to healthy individuals.^34^ And it is believed that the process of tooth decay and gingivitis is largely controlled by a natural defense mechanism in the saliva. The items mentioned above are summarized in Fig. 7.

According to articles that were mentioned above, patients with thalassemia are more likely to develop tooth decay.^35^ One of the causes of this phenomenon is that patients with thalassemia delay their visit to the dentist because they feel that the dentist will not understand the conditions of their systemic disease. Since caries is more likely to occur in patients with thalassemia major due to blood transfusions and poor immune systems. Also, due to the complexity of medical problems, these patients often delay receiving dental services until they can no longer tolerate the pain. In these stages, the caries is so widespread that it usually leads to abscesses and infections in the oral cavity, which may even involve the lymph nodes in the head and neck. In such cases, the main way to prevent the spread of infection in these patients is to eliminate its origin. As a result, they are more prone to tooth extraction, resulting in more loss of teeth in patients with thalassemia than in normal people.^33,35,36^

Some articles have suggested that lower levels of IgA in the saliva of children with thalassemia may be the cause of more caries in them compared to healthy children.^27,37^ In some other studies, endocrine dysfunction in patients with thalassemia is considered to be the main cause of a higher chance of tooth decay in these patients.^26^ Given the spread of thalassemia in Iran, and especially in the northern regions of the country, it seems necessary to establish centers for screening dental caries among these patients.^38^

Dental problems in patients with thalassemia usually approach critical stages due to a lack of timely visits for dental examinations and early diagnosis.^36^ It is recommended that the family dentist system which is also known as dental home in developed countries and is based on regular visits of the dental team to the patient’s place of residence for oral health education and oral health status screening, to be started for patients with thalassemia in early ages.

This identifies the oral problems of these patients in the early stages, and therefore by performing cheaper and simpler treatments not only dental visits would become much more acceptable for these patients who have frequent hospital visits but also will reduce financial costs which benefits the government and insurance companies.

### Suggestions

It is suggested that measures be taken that dental services are easily available to these specific patients. It also appears that routine oral examinations of patients with thalassemia major require special attention in policy-making so that this population group can be routinely evaluated for oral and dental status and treated if necessary.

### Limitations

One of the limitations of this study is that some variables could not be extracted, and subgroup analysis was not possible according to the variables.

## Conclusion

This study showed that thalassemia has a negative effect on the DMFT index, which is an important parameter in oral health status.

## Data Availability

The datasets used and/or analyzed during the current study are available from the corresponding author on reasonable request.
